# The shoulder stiffness scale: a validated tool for monitoring stiffness in arthroscopic rotator cuff reconstructions

**DOI:** 10.1007/s00402-026-06402-4

**Published:** 2026-07-06

**Authors:** Samy Bouaicha, Matthias Biner, Farah Selman, Karl Wieser, Andreas Marc Müller, Laurent Audigé

**Affiliations:** 1 Department of Orthopedics, Balgrist University Hospital, University of Zurich, Zurich, Switzerland; 2 Department of Orthopaedics and Traumatology, University Hospital Basel, Basel, Switzerland; 3 Surgical Outcome Research Center, University of Basel, Basel, Switzerland

**Keywords:** Postoperative shoulder stiffness, Rotator cuff reconstruction, Shoulder stiffness scale, Capsulitis, Frozen shoulder, Shoulder stiffness monitoring

## Abstract

**Background:**

Postoperative shoulder stiffness after arthroscopic rotator cuff repair is common and clinically heterogeneous. Established shoulder scores assess pain and function, but do not specifically isolate stiffness during follow-up. The aim of this study was to validate the newly developed Shoulder Stiffness Scale (SSS) as a pragmatic monitoring tool for postoperative shoulder stiffness after arthroscopic rotator cuff repair (ARCR).

**Methods:**

From a cohort of 973 primary arthroscopic rotator cuff reconstructions, patients without concomitant joint pathology of the same or contralateral arm were included. The new SSS was based on three items related to shoulder pain, subjective and objective ROM ranging from 0 to 10 points. It was collected before surgery, as well as 6 and 12 months postoperatively, along with other pain level parameters, Constant Score (CS), Subjective Shoulder Value (SSV), Oxford Shoulder Score (OSS). Changes of the SSS and its subscales over time, Cronbach's alpha for internal consistency, bottom and ceiling effects were reported. Validity was investigated through SSS associations with other parameters of similar dimension, and functional scores.

**Results:**

The selected cohort with 740 patients showed postoperative shoulder stiffness in 12.7 %. The SSS averaged 5.1 points (SD 2.5) preoperatively, and decreased significantly to mean 2.9 (SD 2.4) and 1.6 (SD 1.9) at 6 and 12 months, respectively (p<0.001). Cronbach's alpha ranged from 0.612 to 0.740 between time points. The SSS showed significant correlation in all three subscales pain, subjective ROM and objective ROM as well as to the functional scores CS, OSS and SSV scores at baseline, 6 months and 12 months with correlation coefficients ranging from -0.302 (low) to -0.649 (moderate) at baseline and from -0.626 (moderate) to -0.719 (high) at 12 months.

**Conclusions:**

The SSS demonstrated acceptable internal consistency and construct validity as a simple clinician-administered tool for monitoring postoperative shoulder stiffness after ARCR. It complemented existing scores by isolating stiffness as a distinct construct and may facilitate day-to-day clinical follow-up and patient communication.

**Level of evidence:**

III.

## Introduction

Adhesive capsulitis and postoperative shoulder stiffness share clinical features such as pain and motion loss, but they are not identical entities. In the postoperative setting, transient stiffness may be part of normal recovery, whereas pathological stiffness reflects a more persistent and clinically relevant deviation from the expected course [1]. Thus, its etiology and pathogenesis are not fully understood and therapeutic options are limited, leaving many affected patients with a long period of intensive shoulder pain. The average duration of the pathology is 18 months with courses of 6–60 months according to the literature [2–4]. Although the exact pathogenesis of the disease remains unclear, a variety of risk factors have been identified: The best-known associated factors were found to be the two endocrine disorders diabetes mellitus and hypothyroidism [5–8]. As the disease is self-limiting in most cases and not everybody with a stiff shoulder is seen by a healthcare professional, valid epidemiological data are limited. The prevalence of idiopathic frozen shoulder was reported to be as high as 5% or 2.4/1000/year, and the development of pathological postoperative stiffness after shoulder surgery ranges from 5 to 33% [2, 9–12]. However, since a limited phase of postoperative shoulder stiffness is part of the natural healing process, especially after rotator cuff surgery, the reported numbers might be falsely low. Further, the lack of a common sense with regard to uniform definition of a pathological stiffness complicates the comparability between scientific reports. The prolonged course of the disease is often associated with an inability to return to work and leisure activities [2, 3, 13].

Although, there is common sense about the three phases of frozen shoulder: freezing, frozen, and thawing, the course of the disease can vary considerably between patients [4]. No simple dedicated instrument has been established for serial monitoring of postoperative shoulder stiffness after ARCR. Established shoulder scores assess broader domains such as pain and function, but do not specifically isolate stiffness as their primary monitoring target [14]. Therefore, the Shoulder Stiffness Scale (SSS) was developed as a brief clinician-administered monitoring tool combining pain, subjective range of motion (ROM) limitation, and objective ROM restriction.

This prospective, multicenter observational study (ARCR_Pred) was conducted to document and predict the safety and effectiveness of arthroscopic rotator cuff repair (ARCR) in a representative patient cohort [15]. Within this framework, we aimed to validate the newly developed SSS in a large ARCR population by examining its change over time, internal consistency, and construct validity. We hypothesized that the SSS would correlate with pain, ROM, and established shoulder scores such as the Constant Score (CS) [16], Oxford Shoulder Score (OSS) [17], and Subjective Shoulder Value (SSV) [18].

## Methods

### ARCR_Pred study setting

Between June 2020 and November 2021, a cohort of 973 primary ARCR patients were prospectively enrolled at the participating centers [15]. Functional and structural outcomes, as well as patient-reported outcome measures (PROMS) and adverse events were recorded at 6-week, 6-, 12- and 24-month follow-up, respectively. Postoperative rehabilitation was generally comparable across centers, consisting of 6 weeks of passive mobilization followed by 6 weeks of active mobilization, although slight between-center variations may have remained.

### Patient baseline characteristics & outcome parameters

For this secondary analysis, we included patients from the ARCR_Pred cohort undergoing primary ARCR regardless of rotator cuff tear severity and excluded those reporting symptomatic concomitant pathology of the ipsilateral or contralateral upper extremity that could confound pain or ROM assessment. Patient demographics, socioeconomic and health-related factors, including the rotator cuff integrity, were documented. Clinical examination and patient-reported subjective outcome parameters at baseline and follow-up at 6 and 12 months included shoulder pain, ROM and strength (CS), as well as the patient-reported Subjective Shoulder Value (SSV) and Oxford Shoulder Score (OSS).

### Shoulder stiffness scale (SSS)

The proposed SSS was composed of 3 parameters: pain (0–3 points), subjective ROM limitation (0–3 points), and objective ROM limitation (0–4 points). The total SSS is obtained by addition of the 3 subscales, ranging from 0 (no stiffness) to 10 points (strong stiffness) (Table 1). The SSS was documented only once by respective attending shoulder and elbow surgeons and assessor clinicians during routine patient examination at baseline prior to surgery, and at 6- and 12-months follow-up.

**Table 1:** Description of the Shoulder Stiffness Scale (SSS)

sss1: Pain	
0 = No pain 1 = Pain at the end of range of motion (ROM) 2 = Pain with light movements (e.g. moving computer mouse) 3 = Pain at rest	
sss2: Subjective ROM limitation	
0 = No subjective ROM limitation 1 = ROM limitation with recreational activities (e.g. sports, gardening) 2 = ROM limitation with physical work 3 = ROM limitation with activities of daily living (e.g. tooth brushing)	
sss3: Objective ROM limitation	
0 = No objective ROM limitation 1 = Passive ER (in adduction) and/or glenohumeral abduction side-to-side difference < 10° 2 = Passive ER (in adduction) and/or glenohumeral abduction side-to-side difference 10–20° 3 = Passive ER (in adduction) and/or glenohumeral abduction side-to-side difference 21–40° 4 = Passive ER (in adduction) and/or glenohumeral abduction side-to-side difference > 40°	

ER = External rotation

The SSS is obtained by addition of the three subscales (ss1 + ss2 + ss3), ranging from 0 to 10 points

Further, a definition for a postoperative shoulder stiffness composite (POSS) outcome was defined for the entire cohort independently of the SSS as follows, whereby at least one of the following conditions had to be met:

1. Any restriction in passive ROM occurring at least 3 months after the operation implying a modification in the usual patient’s care (e.g. physiotherapy or medication, or an intervention requiring manipulation under anesthesia), or:
2. A persisting post-operative restriction at 6 months in passive motion in at least two planes (flexion, abduction, and external rotation in zero-degree abduction). The assessment of the restriction in ROM has been done separately for each plane: Flexion: total motion inferior or equal to 90 degrees or glenohumeral motion (fixed scapula) inferior or equal to 80 degrees. Abduction: total motion inferior or equal to 80 degrees or glenohumeral motion (fixed scapula) inferior or equal to 60 degrees. External rotation in zero-degrees abduction: glenohumeral (fixed scapula) motion inferior or equal to 20 degrees or inferior to 50% of the contralateral side value.

### Data management and statistical analyses

Study data were managed using the REDCap Electronic Data Capture system [19] and exported for variable transformation (including score calculations) and statistical analysis using Intercooled Stata, version 17 (StataCorp LP, College Station, TX, USA).

Baseline patient parameters were tabulated using standard descriptive statistics. The distribution of SSS subscale responses was tabulated and graphed by examination time point. Scale changes over time was assessed by the Friedman’s analysis of variance (ANOVA) for ordered categories (SSS question responses) and repeated-measures ANOVA (SSS value). The internal consistency of the SSS assessment instrument was quantified at each time point by the Cronbach’s alpha (α), whereby a value above 0.70 would be deemed acceptable [20]. In addition, the proportion of patients with a SSS equal to zero (bottom effect) or 10 (ceiling effect) was reported.

The SSS pain parameter was validated with regard to its expected association with other pain questions collected during the ARCR_Pred study:

1. A pain level question as part of the Constant-Murley Score documenting the “Highest pain during activities within 24 hours” transformed to a number rating scale (NRS 0: no pain to 10: maximum pain),
2. Another pain NRS (0: no pain to 10: intolerable pain) documenting the overall “Pain level over the last week”, and
3. One question of the OSS documenting the “Worst pain from shoulder over the past 4 weeks” on a Likert scale (1, None | 2, Mild | 3, Moderate | 4, Severe | 5, Unbearable). The association between the SSS pain parameter with these three measures of pain was investigated at each time point, respectively, using ANOVA and Chi-square test, as well as by Pearson’s correlation coefficients. The SSS subjective and objective ROM parameters were validated with regard to their expected association with subjective patient-reported functional outcomes (SSV and OSS), and objective ROM measurements (passive external rotation at 90° abduction and glenohumeral passive abduction), respectively. We performed ANOVA and calculated Pearson’s correlation coefficients, separately at each examination time point. The overall SSS was examined by scatter plots and Pearson’s correlation with regard to its association with functional parameters CS, OSS and SSV at each time point, respectively.

All analyses were explorative with a significance level set at 0.05. As this was an exploratory secondary validation analysis within the prospective ARCR_Pred cohort, no separate a priori sample size calculation was performed, and all eligible patients with available data were included. Our sample size exceeded published recommendations for validation studies, including those for the assessment of Cronbach’s alpha [21]. Pearson correlation coefficients were used as a pragmatic measure of construct validity, as the total SSS was treated as an approximately continuous summed score in this large cohort.

## Results

### Patient cohort

**Fig. 1:**
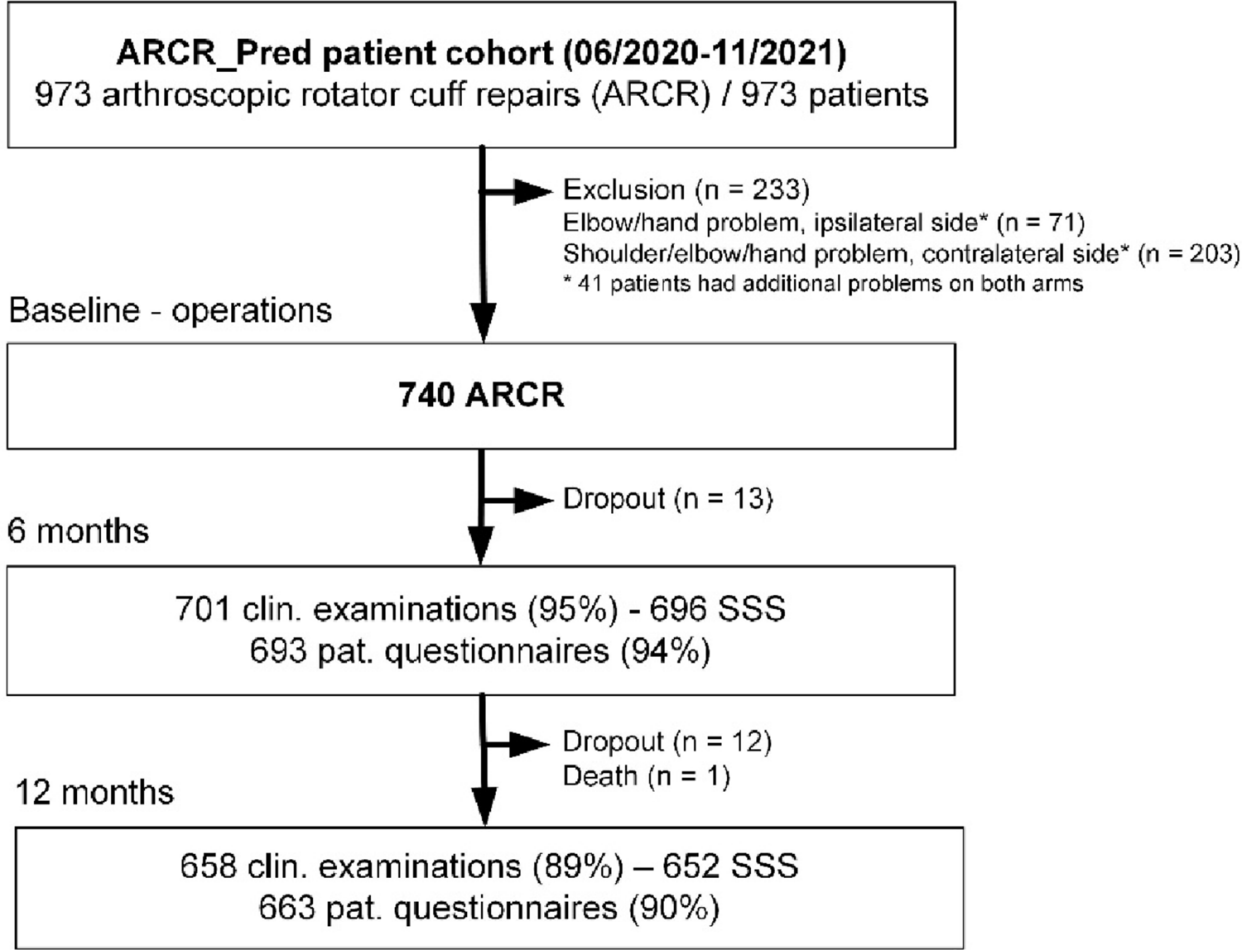
Flowchart of patient selection and follow-up

From the whole ARCR_Pred cohort study, 740 patients (76%) did not report any concomitant joint pathology on the ipsilateral or contralateral arm and were selected for the analysis (Fig. 1). The follow-up rate regarding clinical examination at 6 and 12 months was 95% and 89%, respectively.

The cohort consisted of 269 women (36%) with a mean age at surgery of 56.8 ± 9.5 years and a mean BMI of 26.8 ± 4.5 kg/m^2^ (Table 2). 47% of patients were classified as ASA I, 47% as ASA II, 6% were ASA III and one patient ASA IV. According to surgeon’s judgement to the cause of the shoulder complaint, 26% were purely degenerative and 31% were purely traumatic. 27% of patients had a sole supraspinatus (SSP) tendon tear, 8% sole subscapularis (SSC) tendon tear, and 19% SSP/ISP and SSC tear. 45% were massive tears (2 or more full-thickness tears). Based on the POSS composite outcome, a stiff shoulder was found in 12.7% of cases within the first 6 months postoperatively.

**Table 2:** baseline patient characteristics

Baseline parameters	*n*(%)	mean (SD)
Age at surgery	740	56.8 (9.5)
Gender (n,%)		
Female	269 (36)	
Male	471 (64)	
ASA classification (n,%)		
I	347 (47)	
II	345 (47)	
III	47 (6)	
IV	1	
Body Mass Index (BMI)	740	26.8 (4.5)
One or more comorbidities (n,%)		
No	385 (52)	
Yes	355 (48)	
History of frozen shoulder on any side (n,%)		
No	727 (98)	
Yes	13 (2)	
Surgeon’ judgement as to the cause of the shoulder complaint		
Purely degenerative	193 (26)	
More degenerative than traumatic	127 (17)	
More traumatic than degenerative	187 (25)	
Purely traumatic	233 (31)	
RC tendon(s) ruptured (n,%)		
ISP	2 (0)	
SSC	58 (8)	
SSP	203 (27)	
SSP & ISP	176 (24)	
SSP & ISP & SSC	143 (19)	
SSP & SSC	158 (21)	
RC tear severity (intra-operative) (n,%)		
Partial tear	115 (16)	
Single full tear	183 (25)	
Two or three tendons (only one full)	109 (15)	
Massive tear (Gerber et al.)	333 (45)	

### Descriptive statistics and change over time

**Fig. 2:**
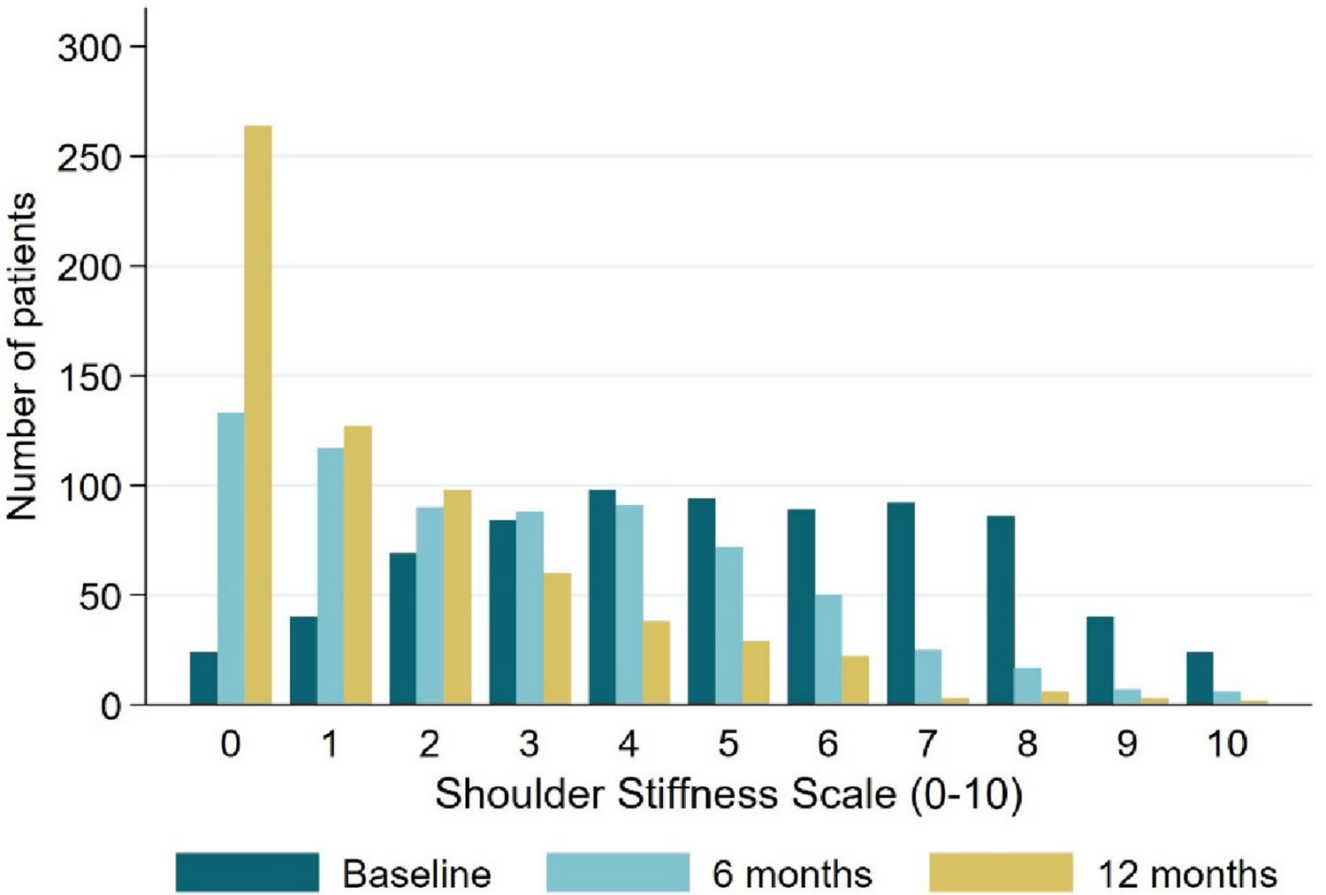
Distribution of the Shoulder Stiffness Scale at baseline, 6 months and 12 months

The SSS showed an internal consistency with Cronbach’s alpha of 0.612, 0.740 and 0.739 at baseline, 6-month and 12-month time points, respectively. The SSS decreased significantly over time from a mean 5.1 points at baseline to 1.6 points at 12-month follow-up (Fig. 2; Table 3). The proportion of patients with a SSS equal to zero (bottom effect) at baseline, 6 and 12 months was 3%, 19% and 41%, respectively. That of patient with a maximum SSS of 10 points did not exceed 3% as observed at baseline. The SSS is reflected by the changes of its subscales. 93% of patients had pain at baseline (pain at the end of ROM, with light movement or at rest). At 12-months follow-up 71% were pain free, 23% had only pain at the end of ROM. 81% of the cohort had subjective limitation of the ROM at baseline. At 12-months follow-up 28% persisted complaining about limited ROM. Objectively 69% of patients had ROM limitation at baseline and 48% at 12-months follow-up.

**Table 3:** Distribution and change of Shoulder Stiffness Scale (SSS) parameters from baseline to 6- and 12-month follow-up examination

SSS parameters	Baseline	6 months	12 months	
	*n* (%)	*n* (%)	*n* (%)	*P*-value
Pain				< 0.001
No pain	49 (7)	370 (53)	466 (71)	
Pain at the end of range of motion (ROM)	246 (33)	254 (36)	151 (23)	
Pain with light movements	229 (31)	45 (6)	24 (4)	
Pain at rest	216 (29)	30 (4)	14 (2)	
Subjective ROM limitation				< 0.001
No subjective ROM limitation	144 (19)	318 (45)	472 (72)	
ROM limitation with recreational activities	153 (21)	210 (30)	116 (18)	
ROM limitation with physical work	147 (20)	107 (15)	48 (7)	
ROM limitation with activities of daily living	296 (40)	64 (9)	18 (3)	
Objective ROM limitation				< 0.001
No objective ROM limitation	233 (31)	204 (29)	337 (52)	
Pass. ER add. / GH abd. diff. <10°	146 (20)	153 (22)	136 (21)	
Pass. ER add. / GH abd. diff. 10–20°	211 (29)	224 (32)	142 (22)	
Pass. ER add. / GH abd. diff. 21–40°	97 (13)	87 (12)	32 (5)	
Pass. ER add. / GH abd. diff. >40°	53 (7)	30 (4)	7 (1)	
Shoulder Stiffness Scale (SSS 0–10)	740	696	652	< 0.001
Mean (SD)	5.1 (2.5)	2.9 (2.4)	1.6 (1.9)	

Pass. ER add. = Passive External Rotation in adduction; GH abd. Diff = Glenohumeral abduction difference between sides; P-value = Friedman’s ANOVA test p-value (ordered categories) and Repeated-measures ANOVA p-value (SSS)

### SSS pain subscale

The SSS correlated significantly with the three measures of pain (highest pain in the last 24 h, highest pain level and degree of worst pain in the last week) at baseline, 6- and 12-month follow-up (Table 4).

**Table 4:** Association between the pain parameter of the Shoulder Stiffness Scale with three measures of pain at baseline, 6- and 12-month follow-up examination

	Categories of Shoulder Stiffness Scale pain parameter^1^					
	None	End of ROM	Light movements	Rest		Correlation
Time points and pain parameters	n (%)	n (%)	n (%)	n (%)	p-value^2^	Coefficient^3^
Baseline						
Highest pain − 24 hours^4^	49	246	229	216	< 0.001	0.385
mean (SD)	3.5 (2.4)	4.7 (2.1)	5.4 (2.2)	6.5 (1.9)		
median (range)	3.3 (0.0 to 8.7)	4.7 (0.0 to 10.0)	5.3 (0.0 to 10.0)	6.7 (2.0 to 10.0)		
Pain level - last week^5^	49	246	229	216	< 0.001	0.283
mean (SD)	4.9 (2.7)	5.0 (2.2)	5.8 (2.0)	6.5 (1.9)		
median (range)	5.0 (0.0 to 10.0)	5.0 (0.0 to 10.0)	6.0 (1.0 to 10.0)	7.0 (1.0 to 10.0)		
Worst pain - past 4 weeks^6^					< 0.001	0.247
None	2 (4)	3 (1)		1 (0)		
Mild	7 (14)	30 (12)	13 (6)	4 (2)		
Moderate	18 (37)	81 (33)	75 (33)	47 (22)		
Severe	20 (41)	122 (50)	130 (57)	139 (64)		
Unbearable	2 (4)	10 (4)	11 (5)	25 (12)		
6 months						
Highest pain − 24 hours^4^	370	254	44	30	< 0.001	0.541
mean (SD)	0.9 (1.6)	2.5 (1.9)	3.5 (1.6)	5.0 (2.1)		
median (range)	0.0 (0.0 to 10.0)	2.0 (0.0 to 9.3)	3.3 (0.0 to 6.7)	5.0 (1.3 to 9.3)		
Pain level - last week^5^	364	246	44	27	< 0.001	0.517
mean (SD)	1.6 (1.7)	3.3 (2.1)	4.9 (2.0)	5.3 (2.2)		
median (range)	1.0 (0.0 to 10.0)	3.0 (0.0 to 9.0)	5.0 (1.0 to 9.0)	5.0 (1.0 to 9.0)		
Worst pain - past 4 weeks^6^					< 0.001	0.510
None	112 (31)	17 (7)				
Mild	200 (55)	89 (36)	9 (20)	3 (11)		
Moderate	40 (11)	105 (43)	25 (57)	10 (37)		
Severe	11 (3)	30 (12)	9 (20)	14 (52)		
Unbearable	1 (0)	5 (2)	1 (2)			
12 months						
Highest pain − 24 hours^4^	466	151	24	14	< 0.001	0.609
mean (SD)	0.5 (1.2)	2.4 (2.1)	4.2 (2.4)	5.6 (2.1)		
median (range)	0.0 (0.0 to 10.0)	1.3 (0.0 to 10.0)	4.7 (0.0 to 8.7)	5.3 (2.0 to 9.3)		
Pain level - last week^5^	461	147	23	14	< 0.001	0.606
mean (SD)	1.0 (1.4)	3.0 (2.0)	4.7 (2.2)	6.2 (2.0)		
median (range)	0.0 (0.0 to 8.0)	2.0 (0.0 to 9.0)	5.0 (0.0 to 8.0)	6.0 (3.0 to 10.0)		
Worst pain - past 4 weeks^6^					< 0.001	0.553
None	271 (59)	16 (11)	3 (13)			
Mild	149 (32)	65 (44)	3 (13)	1 (7)		
Moderate	30 (7)	53 (36)	9 (39)	5 (36)		
Severe	11 (2)	12 (8)	8 (35)	7 (50)		
Unbearable		1 (1)		1 (7)		

SD = Standard Deviation

1 Categories of the pain parameters were defined as: 0, No pain | 1, Pain at the end of range of motion [End of ROM] | 2, Pain with light movements (e.g. moving computer mouse) [Light movements] | 3, Pain at rest [Rest]

2 P-value = ANOVA test p-value (number rating scales) and Chi-square test p-value (ordered categories)

3 Correlation Coefficient = Pearson’s correlation coefficient

4 “Highest pain during activities within 24 hours” on a number rating scale (NRS 0: no pain to 10: maximum pain)

5 “Pain level over the last week” on an NRS (0: no pain to 10: intolerable pain)

6 “Worst pain from shoulder over the past 4 weeks” on a Likert scale

At *baseline*, there was a significant association between the SSS pain parameter, and the highest pain experienced during activities within the past 24 h (*p* < 0.001). The correlation coefficient was moderate (*r* = 0.385). Patients reporting pain at rest had a higher mean pain score (6.5 ± 1.9) compared to those reporting pain with light movements (5.4 ± 2.2) or only at the end of ROM (4.7 ± 2.1). A similar pattern was observed for the pain level over the last week (*p* < 0.001, *r* = 0.283), with higher pain scores correlating with increased SSS pain parameter scores. For the worst pain experienced over the past 4 weeks, the association was significant (*p* < 0.001), with the majority of patients reporting moderate to severe pain corresponding to higher SSS pain scores (*r* = 0.247).

At *6 months*, the SSS pain parameter continued to show significant associations with the highest pain during activities within 24 h (*p* < 0.001, *r* = 0.541). Patients with pain at rest had a substantially higher mean pain score (5.0 ± 2.1) compared to those experiencing pain with light movements (3.5 ± 1.6) or only at the end of the ROM (2.5 ± 1.9). The pain level over the last week remained significantly associated with the SSS pain parameter (*p* < 0.001, *r* = 0.517). Similarly, the worst pain over the past 4 weeks showed a significant association with the SSS pain parameter (*p* < 0.001, *r* = 0.510), with severe pain categories becoming more prevalent among patients with higher SSS pain scores.

At *12 months*, the associations between the SSS pain parameter and the pain measures were further strengthened. The correlation with the highest pain during activities within 24 h increased (*p* < 0.001, *r* = 0.609), with the highest mean pain score observed in patients with pain at rest (5.6 ± 2.1). The pain level over the last week also showed a stronger correlation with the SSS pain parameter (*p* < 0.001, *r* = 0.606). The association with the worst pain over the past 4 weeks remained significant (*p* < 0.001, *r* = 0.553), with a notable increase in severe pain reports among those with higher SSS pain scores.

### SSS subjective ROM subscale

There were significant associations between the subjective ROM parameter of the SSS and patient-reported functional outcomes (OSS and SSV) (Table 5).

**Table 5:** Association between the subjective ROM parameter of the Shoulder Stiffness Scale with patient-reported subjective functional parameters at baseline, 6- and 12-month follow-up examination

	Categories of SSS subjective ROM limitation parameter ^1^					
	None	Rec. activities	Physical work	ADL		Correlation
Time points and pain parameters	n (%)	n (%)	n (%)	n (%)	p-value	Coefficient
Baseline						
Oxford Shoulder Score (0–48)	144	153	147	296	< 0.001	-0.445
mean (SD)	32 (8)	31 (8)	30 (7)	23 (8)		
median (range)	33 (11 to 48)	31 (9 to 47)	31 (12 to 44)	23 (2 to 42)		
Subjective Shoulder Value (%)	144	153	147	296	< 0.001	-0.312
mean (SD)	54 (16)	53 (18)	49 (18)	40 (20)		
median (range)	60 (5 to 90)	50 (5 to 100)	50 (6 to 90)	40 (0 to 95)		
6 months						
Oxford Shoulder Score (0–48)	316	205	101	58	< 0.001	-0.552
mean (SD)	44 (5)	40 (6)	35 (8)	32 (8)		
median (range)	45 (19 to 48)	41 (16 to 48)	35 (16 to 48)	32 (10 to 47)		
Subjective Shoulder Value (%)	315	205	101	58	< 0.001	-0.502
mean (SD)	84 (12)	75 (16)	66 (16)	59 (18)		
median (range)	85 (20 to 100)	80 (15 to 100)	70 (10 to 100)	60 (20 to 90)		
12 months						
Oxford Shoulder Score (0–48)	466	113	48	17	< 0.001	-0.622
mean (SD)	46 (4)	41 (7)	36 (8)	27 (10)		
median (range)	47 (23 to 48)	43 (14 to 48)	37 (13 to 48)	29 (8 to 46)		
Subjective Shoulder Value (%)	465	113	48	17	< 0.001	-0.592
mean (SD)	91 (10)	81 (14)	67 (18)	55 (22)		
median (range)	95 (30 to 100)	85 (40 to 100)	70 (30 to 95)	55 (20 to 100)		

1 Categories of the subjective range of motion parameters were defined as presented in Table 1;

P-value = analysis of variance (ANOVA) test p-value; Correlation Coefficient = Pearson’s correlation coefficient; SD = Standard Deviation

*At baseline*, a significant inverse association was observed between the SSS subjective ROM parameter and the OSS (*p* < 0.001, *r*=-0.445). Patients with no reported ROM limitation had the highest OSS (32 ± 8), while those reporting limitations in ADL had the lowest OSS (23 ± 8). Similarly, the SSV also demonstrated a significant inverse association with the SSS subjective ROM parameter (*p* < 0.001, *r*=-0.312). Patients with no ROM limitations reported a higher SSV (54% ± 16%), whereas those with ADL limitations reported a lower SSV (40% ± 20%).

*At 6 months*, these associations were further increasing. The OSS remained significantly associated with the SSS subjective ROM parameter (*p* < 0.001, *r*=-0.552), with the highest score observed in patients with no ROM limitations (44 ± 5) and the lowest in those with ADL limitations (32 ± 8). The SSV also showed a significant inverse association with the SSS subjective ROM parameter at this time point (*p* < 0.001, *r*=-0.502), with patients without ROM limitations reporting higher SSV (84% ± 12%) compared to those with ADL limitations (59% ± 18%).

By *12 months*, the association between the SSS subjective ROM parameter and both the OSS and the SSV were even more pronounced. The OSS was highly correlated with the SSS subjective ROM parameter (*p* < 0.001, *r*=-0.622), with patients reporting no ROM limitations achieving the highest OSS (46 ± 4) and those with ADL limitations the lowest (27 ± 10). The SSV also maintained a strong inverse association (*p* < 0.001, *r*=-0.592), with SSV scores being highest in patients with no ROM limitations (91% ± 10%) and lowest in those with ADL limitations (55% ± 22%).

### SSS objective ROM subscale

A further significant inverse relationship was found between the objective ROM parameter of the SSS and objective ROM measurements (Table 6).

**Table 6:** Association between the objective ROM parameter of the Shoulder Stiffness Scale with ROM in passive external rotation at 90° abduction and glenohumeral passive abduction at baseline, 6- and 12-month follow-up examination

	Categories of SSS objective ROM limitation parameter						
	None	Diff < 10°	Diff 10–20°	Diff 21–40°	Diff > 40°		Correlation
Time points and pain parameters	n (%)	n (%)	n (%)	n (%)	n (%)	p-value	Coefficient
Baseline							
Passive ext. rot. at 90° abd. (°)	233	145	211	97	52	< 0.001	-0.449
mean (SD)	74 (22)	66 (26)	60 (26)	48 (29)	24 (28)		
median (range)	80 (0 to 100)	70 (0 to 100)	70 (0 to 100)	50 (0 to 90)	15 (0 to 90)		
GH passive abduction (°)	233	146	211	97	52	< 0.001	-0.284
mean (SD)	91 (13)	89 (19)	89 (17)	81 (21)	67 (26)		
median (range)	90 (40 to 120)	90 (30 to 120)	90 (40 to 120)	80 (30 to 120)	70 (10 to 120)		
6 months							
Passive ext. rot. at 90° abd. (°)	202	150	223	84	30	< 0.001	-0.433
mean (SD)	75 (17)	74 (19)	65 (20)	50 (25)	35 (25)		
median (range)	80 (10 to 100)	80 (10 to 100)	70 (0 to 100)	60 (0 to 90)	40 (0 to 90)		
GH passive abduction (°)	202	151	224	86	30	< 0.001	-0.272
mean (SD)	92 (11)	94 (17)	89 (16)	80 (22)	72 (23)		
median (range)	90 (40 to 120)	90 (30 to 120)	90 (50 to 120)	80 (30 to 120)	70 (20 to 120)		
12 months							
Passive ext. rot. at 90° abd. (°)	331	136	141	32	7	< 0.001	-0.429
mean (SD)	80 (14)	76 (17)	69 (19)	50 (23)	27 (22)		
median (range)	80 (20 to 100)	80 (10 to 100)	70 (10 to 100)	50 (10 to 90)	30 (0 to 60)		
GH passive abduction (°)	332	136	141	32	7	< 0.001	-0.160
mean (SD)	95 (12)	98 (16)	92 (15)	86 (20)	73 (14)		
median (range)	90 (60 to 120)	90 (60 to 120)	90 (50 to 120)	90 (50 to 120)	80 (50 to 90)		

1 Categories of the objective range of motion parameters were defined as presented in Table 1; Diff = difference between operated and contralateral side

P-value = analysis of variance (ANOVA) test p-value; Correlation Coefficient = Pearson’s correlation coefficient; SD = Standard Deviation;

GH = gleno-humeral; ext.rot. = external rotation; abd. = abduction

At *baseline*, the SSS objective ROM parameter was significantly associated with passive ER at 90° ABD (*p* < 0.001, *r*=-0.449). Patients with no ROM limitation had the highest mean ER (74° ± 22°), while those with a > 40° difference in ROM between the operated and contralateral side had the lowest mean ER (24° ± 28°). Similarly, the glenohumeral passive ABD also demonstrated a significant inverse association with the SSS objective ROM parameter (*p* < 0.001, *r*=-0.284), with the highest mean ABD observed in patients with no ROM limitation (91° ± 13°) and the lowest in those with a > 40° difference (67° ± 26°).

At *6 months*, these associations remained significant. The SSS objective ROM parameter continued to correlate inversely with passive ER at 90° ABD (*p* < 0.001, *r*=-0.433), with mean ER decreasing as ROM limitation increased (no limitation: 75° ± 17°, > 40° difference: 35° ± 25°). Glenohumeral passive ABD also maintained its significant inverse association with the SSS objective ROM parameter (*p* < 0.001, *r*=-0.272), with mean ABD decreasing from 92° ± 11° in patients with no limitation to 72° ± 23° in those with the greatest ROM limitation.

At *12 months*, the associations between the SSS objective ROM parameter and both ROM measurements persisted. The inverse correlation between the SSS objective ROM parameter and passive ER at 90° ABD remained significant (*p* < 0.001, *r*=-0.429), with patients showing no ROM limitation achieving a mean ER of 80° ± 14°, compared to 27° ± 22° in those with a > 40° difference. The association with gleno-humeral passive ABD was still significant but weaker (*p* < 0.001, *r*=-0.160), with the highest mean ABD seen in patients with no ROM limitation (95° ± 12°) and the lowest with a > 40° difference (73° ± 14°).

### Association of SSS with functional scores

**Fig. 3:**
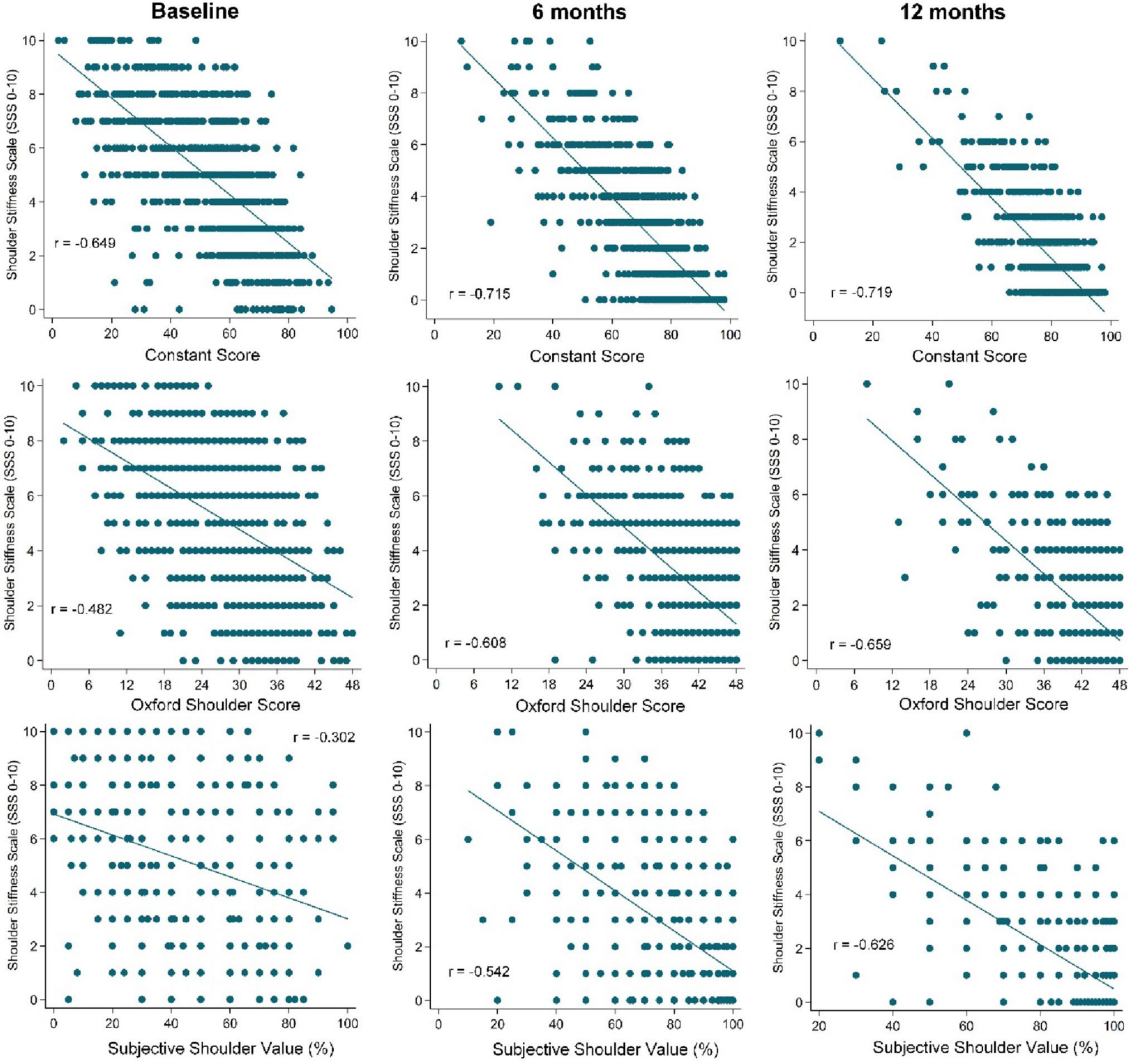
Scatter plots of association between the Shoulder Stiffness Scale and functional scores at baseline, 6 months and 12 months

The SSS showed significant negative association with functional CS, OSS and SSV scores at baseline, 6 months, and 12 months (Fig. 3) with correlation coefficients ranging from − 0.302 (low) to -0.649 (moderate) at baseline and from − 0.626 (moderate) to -0.719 (high) at 12 months.

## Discussion

Although frozen shoulder in general and postoperative stiffness in particular are among the most common and long-lasting shoulder problems [9], there have been no efforts to quantify this entity and thus make it accessible for standardized monitoring. One possible explanation for this phenomenon is probably that, despite repeated attempts to reach a scientific consensus, the criteria for the diagnosis of stiff shoulder continue to be very heterogeneous [22]. Further, the course of postoperative frozen shoulder is very heterogeneous in terms of severity and duration [9, 13]. This is the case despite the disease pattern typically following the three phases of freezing, frozen, and thawing [3]. For this reason, the SSS was developed as a simple composite tool based on pain, subjective ROM, and objective ROM. At present, no categorical interpretation of SSS values, such as mild, moderate, or severe stiffness or phase-specific thresholds, could be recommended.

Since the perception of a stiff shoulder is very subjective and can vary considerably between individuals, two subjective and only one objective parameter was used to assess the degree of severity. To validate the individual parameters of this new tool, the corresponding but independently collected clinical findings of this large-scale multicenter study were used. We could show adequate internal consistency of the SSS, highlighting that its subscales measure a similar dimension of shoulder stiffness. The lower baseline Cronbach’s alpha may reflect the more heterogeneous preoperative symptom profile, whereas postoperative stiffness may emerge as a more distinct clinical construct during follow-up. While no ceiling effect was observed, a bottom effect is noted at follow-up due to the majority of patients recovering well from their rotator cuff repair. The SSS therefore can be seen as an instrument to monitor remaining patients showing stiffness patterns.

Our analysis was able to confirm the hypothesis that the SSS parameters correlate with the data points of the clinical scores and can therefore be applied independently.

The good correlation of the SSS with frequently used shoulder scores such as the CS (*r* = -0.715/-0.719) or the OSS (*r* = -0.608/-0.659) at both 6 and 12 months shows that the stiffness in the sense of capsulitis measured by the SSS represented a useful surrogate for overall shoulder function in the postoperative course. Thus, the SSS can be used in the future for a simple assessment of frozen shoulder and can also be used to monitor it over the course of the disease. Our findings also provide the basis for further investigations, for example to answer the question of whether a typical SSS value can be assigned to the different phases of frozen shoulder, or if the composition of the SSS in idiopathic frozen shoulder and posttraumatic stiffness differ. The POSS-positive subgroup is clinically relevant and future studies should specifically investigate SSS trajectories in these patients.

A major limitation of this investigation is that the SSS was only collected only once by various investigators in the context of an observational cohort, and only at three points in time, baseline, 6 and 12 months. These data however reflect a pragmatic setting and what should be expected in routine practice. The present validation applies to postoperative stiffness after ARCR. Applicability to idiopathic frozen shoulder or other stiffness entities remains hypothetical and should be evaluated separately. As the SSS includes objective range of motion (ROM) measurements recorded only once per visit by different surgeons and assessors across centers, interobserver and intraobserver variability may have influenced the results. Responsiveness of the SSS, including measures such as effect size and minimal clinically important difference, was not assessed in the present study and should be investigated in future studies. In addition, no clinical cut-offs for interpretation of SSS values have yet been established. Moreover, as patients were recruited at specialized centers, selection bias cannot be excluded and generalizability to other clinical settings may be limited. Nevertheless, it is primarily a monitoring tool for shoulder pain and limited movement, which can be recorded regardless of the clinical background.

## Conclusion

The SSS demonstrated acceptable internal consistency and construct validity as a simple clinician-administered tool for monitoring postoperative shoulder stiffness after ARCR. Further studies should evaluate its applicability to other forms of shoulder stiffness and its potential role in identifying different stiffness patterns and guiding treatment.

## Data Availability

Following a period of embargo of at least 2 years after the end of the study in September 2024, metadata describing the type, size, and content of the dataset will be published along with the study protocol on the open repository Zenodo (https://zenodo.org/). Researchers wishing to access the full dataset will be able to file a request with the Data Access Committee of the Medical Faculty of the University of Basel (MF-DAC – email: med-dac@unibas.ch). The MF-DAC will act as an independent assessor of the request and grant access to the dataset if all ethical, legal, and scientific conditions are met.
